# Endoanchors under 3D image fusion for a type IA endoleak after EVAR

**DOI:** 10.1002/ccr3.2033

**Published:** 2019-02-06

**Authors:** Giovanni Tinelli, Francesca De Nigris, Fabrizio Minelli, Roberto Flore, Angelo Santoliquido, Yamume Tshomba

**Affiliations:** ^1^ Vascular Surgery Unit Fondazione Policlinico Univeristario A. Gemelli IRCCS, Roma ‐ Università Cattolica del Sacro Cuore Rome Italy; ^2^ Internal Medicine Fondazione Policlinico Univeristario A. Gemelli IRCCS, Roma ‐ Università Cattolica del Sacro Cuore Rome Italy

**Keywords:** endoanchors, fusion imaging, innovative biotechnologies, personalized medicine

## Abstract

The Heli‐FX technique for type IA EL under 3D‐IF proved to be accurate in terms of EL channel vision and correct endoanchors deployment. The EL volume rendering constant view allowed a precise anchors fixation at the EL channel. 3D‐IF confirmed to be a valid help in orientation and navigation during endovascular aortic procedure.

## 1 INTRODUCTION

Complications at the aortic neck represent one of the remaining challenges of endovascular aneurysm repair (EVAR).^1^, ^2^, ^3^ In particular, type IA endoleak (EL) is associated with a considerably higher risk of aneurysm rupture and therefore necessitates prompt and definitive treatment.^4^ Type IA EL discovered during follow‐up is usually caused by graft migration in a previous hostile aortic neck and/or aortic neck dilatation.^4^ Proximal extension cuffs, fenestrated endografts, the chimney technique, bare metal stents, and external banding have been employed to treat this complication.^5^, ^6^, ^7^, ^8^, ^9^, ^10^

More recently, the Heli‐FX endoanchor System (Medtronic, Santa Rosa, CA, USA) has been used to prevent proximal neck complications in patients with challenging neck anatomy and to treat these complications when they arise.^11^

This system is a helical endovascular suture device intended to provide fixation of an endograft to the aortic wall. According to the instructions for use from the manufacturer, each helically shaped 4.5‐mm‐length, 3‐mm‐diameter EndoAnchor is implanted serially around the circumference of the endograft in the proximal aortic neck.^12^ After the identification of the leak channel, the procedure is completed creating an adjunctive “suture line” in correspondence of the leak. Literature demonstrated satisfactory short‐term success with endoanchors.^11^, ^13^, ^14^

Today's hybrid operating rooms (HOR) facilitate complex and very accurate interventions with advanced radiographic imaging, such as 3‐dimensional image fusion (3D‐IF) of preoperative computed tomography (CT) images with intraoperative fluoroscopy images.^15^ The 3D volume rendering (VR) image overlay fused with 2D fluoroscopy enabled procedure performance with constant road mapping of the aortic wall and the origin of the aortic branches to improve intravascular orientation. This imaging technique is recognized to decrease radiation exposure, procedure time, and contrast usage.^16^, ^17^

This case report describes the treatment of a type IA EL with endoanchors deployment using support with 3D‐IF. The patient gave consent for the publication of the report, and the Institutional Review Board approved it.

## 2 CASE REPORT

An 89‐year‐old man was previously treated with EVAR for an infrarenal aortic aneurysm. A Zenith® LP (Cook Medical, Bloomington, IN, USA; graft diameter 28 mm) was previously used in an aortic neck of 23 mm diameter and 17 mm length. In the follow‐up, CT imaging revealed a type IA EL with aortic neck dilatation (30 mm) and a stent graft migration of 8 mm below the left renal artery.

**Figure 1:**
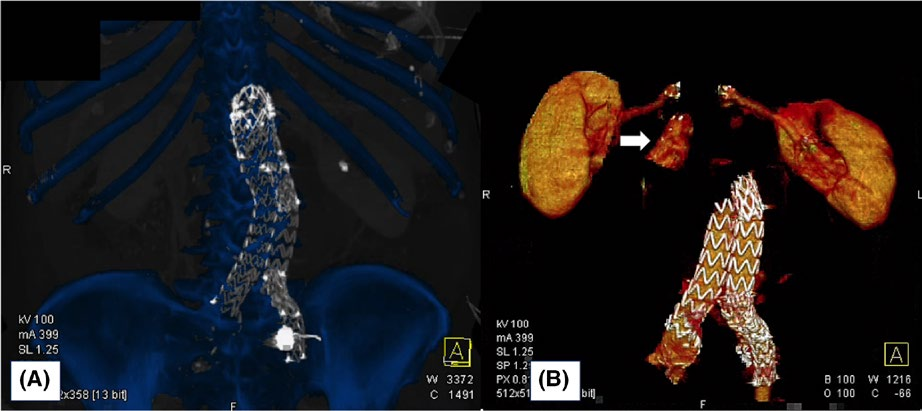
Bone and stent graft scaffolding volume rendering (VR) (A). VR of the type IA endoleak, renal arteries (B)

**Figure 2:**
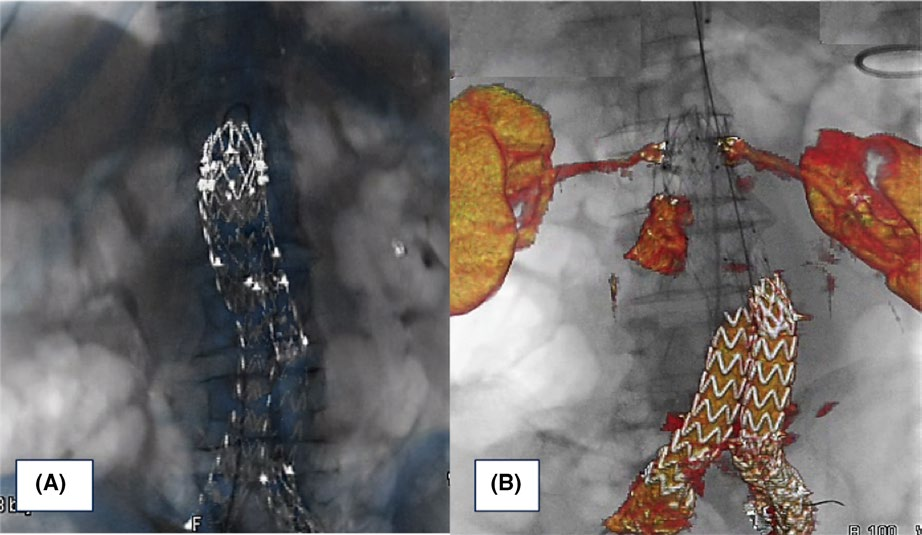
Moment of fusion with stent graft landmarks in AP projection (A). 3D‐IF guidance during the procedure: renal arteries and type IA endoleak VR on fluoroscopy (B)

We decided to treat the type IA EL with a proximal cuff stent graft relining and endoanchor suture using 3D‐IF guidance. In our HOR (Artis Zeego; Siemens Healthcare GmbH, Forchheim, Germany), the procedure was performed under general anesthesia with a bilateral percutaneous approach. The preoperative planning was performed on our 3D workstation (Leonardo, Healthcare Sector, Siemens AG, Forchheim, Germany), producing the previous 3D stent graft scaffolding and 3D vessels volume rendering, which included renal arteries and the type IA EL channel (Figure 1A,B). The fusion technique was performed to align the stent graft scaffolding VR to the live stent graft fluoroscopy in two projections: the antero‐posterior and lateral views. This overlapping was obtained with stiff guides in place to offset arterial stretching (Figure 2A). After this procedure, we switched to the 3D vessels VR to obtain the final roadmap view for the 2D fluoroscopy (Figure 2B).

Using 3D‐IF guidance, we deployed a proximal stent graft cuff (Endurant II, Medtronic, Santa Rosa, CA; graft diameter 36 mm).

**Figure 3:**
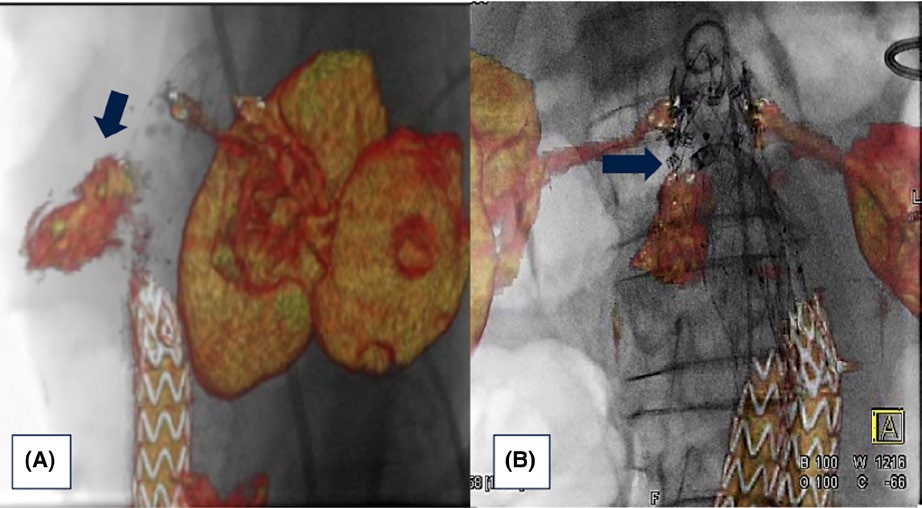
Best intraoperative projection of type IA endoleak channel (A). Positioning and deployment of the Heli‐FX endoanchors in the specific origin of the endoleak for the “endosuture line” (B)

In agreement with the instructions for use, we used Heli‐FX endoanchors to fix the new stent graft to the aortic wall just below the renal arteries. We deployed six endoanchors circumferentially on the cuff in the four quadrants (30° RAO, 30° LAO and 90°C‐arm angulation) and then created a “suture line” along the leak channel using the 3D‐IF view (Figure 3A‐B).

The total duration of the IF technique was 6 minutes, and the total procedure time was 34 minutes (12 minutes fluoroscopy time). No contrast was used during the procedure, and the radiation dose measured by the dose area product (DAP) was 8.3 Gy cm^2^. The predischarge CT showed the resolution of type IA EL, renal arteries patency, and the exact positioning of the endoanchors.

## 3 DISCUSSION

Suboptimal sealing is a major determinant of EVAR failure, including in follow‐up due to the progression of atherosclerotic aortic disease. Graft migration and type IA EL could result from a compromised seal or fixation failure of the proximal aortic neck, particularly with necks that are short, angulated, large in diameter, conical, or those with significant mural thrombus or calcium.^18^

Endovascular redo of a previously treated aorta, typically for an abdominal aortic aneurysm, has become not uncommon procedure with challenging technical aspects.^19^ Considering the technical evolvement of endovascular tools, most of these reinterventions were performed by endovascular means.

This case confirms that endoanchors placement serves as a viable treatment option for type IA EL following EVAR failure. Aortic extender cuffs were used in conjunction with endoanchors when a significant length of the aortic neck was present between the proximal margin of the endograft and the lowest renal artery.^1^, ^20^

Accurate preoperative planning is a critical issue in endovascular procedures, especially in redo endovascular aortic surgery.^21^

Correct endoanchors deployment represents the key point of technical success in creating a new sealing. In accordance with the instructions for use from the manufacturer, we provided a circumferential anchoring (six anchors for an aortic neck diameter ≥30 mm; four for a diameter ≤29 mm) of the stent to the aortic wall. In the second step, we treat the type IA EL with a “suture line” along the leak channel. This technical deployment is feasible with a correct and critical C‐arm positioning for proper spacing, visualization, and implantation. However, leak channel identification is often not very accurate in 2D fluoroscopy.

3D‐IF support in endovascular reinterventions can enable the safe alignment of anatomical structures shown in VR with 2D fluoroscopy, which can be accurately overlapped with landmarks from the previously placed stent graft, as was demonstrated in this case. In redo endovascular procedures, the endograft scaffolding showed several landmarks to improve the volume alignment in the fusion technique, thus making it more accurate.

Precise EndoAnchor appositioning was achieved at the origin of the EL with a good result considering the relative difficulty in orientation and navigation of the proximal cuff device. Of course, EndoAnchor deployment along the EL channel could be placed in fluoroscopy with precise degrees of angulation obtained in the planning study using preoperative CT; however, the situation may require further exposure to X‐ray radiography and the use of contrast to confirm the EL. The major advantage of 3D‐IF is its capability of overcoming the known limitations of 2D angiographic roadmap registration.^22^ The fusion technique provides an accurate visualization of compromised anatomy in need of reintervention. This imaging technique has been shown in complex endovascular procedures to reduce procedure time and limit the use of contrast media and radiation exposure.^16^, ^22^ Therefore, we favor the FI approach in reinterventions because of its potential to significantly decrease radiation exposure while facilitating greater anatomical and procedural visualization. Zero‐contrast reintervention procedures, such as zero‐contrast EVARs, have been reported in literature with FI 3D road mapping.^23^, ^24^ This is an important development because patients who undergo endovascular procedures are very frequently exposed to follow‐up X‐ray examinations and secondary procedures, ultimately increasing their radiation and contrast exposure.

Redo endovascular aortic procedures are technically demanding and carry increased risks. Improving experience and technology, such as fusion imaging, should mitigate some of this risk and are recommended.^19^, ^25^

## 4 CONCLUSION

Redo endovascular aortic procedures are technically demanding. Accurate planning and intraoperative imaging support are essential. The 3D‐IF technique represents a safe and effective imaging tool that is also capable of guiding precise EndoAnchor deployment with the goal of successfully achieving strong fixation and a durable seal of the proximal aortic neck. The further combination of interesting and useful endovascular techniques is warranted to provide a tailored strategy for each patient.

## CONFLICT OF INTEREST

Declaration of Conflicting Interests: nothing to declare.

## AUTHOR CONTRIBUTION

GT, FM, FDN, and YT: involved in conception and design. GT, FM, and FDN: acquired the data. GT, FM, AS, and RF: analyzed and interpreted the data. GT, FM, FDN, RF, and YT: drafted the article. GT, AS, and YT: critically revised the manuscript. GT, FM, FDN, RF, AS, and YT: approved the manuscript.
